# Species and genetic diversity are not congruent in fragmented dry grasslands

**DOI:** 10.1002/ece3.4791

**Published:** 2018-12-12

**Authors:** Christoph Reisch, Christoph Schmid

**Affiliations:** ^1^ Institute of Plant Sciences University of Regensburg Regensburg Germany; ^2^ Research Unit Comparative Microbiome Analysis German Research Center for Environmental Health Neuherberg Germany

**Keywords:** biodiversity, covariation, fragmentation, island biogeography, SGDC

## Abstract

Biological diversity comprises both species diversity (SD) and genetic diversity (GD), and it has been postulated that both levels of diversity depend on similar mechanisms. Species‐genetic diversity correlations (SGDC) are therefore supposed to be generally positive. However, in contrast to theory, empirical data are contradictory. Furthermore, there is a pronounced lack of multispecies studies including also the ecological factors potentially driving species and genetic diversity. We analyzed the relationship between the species diversity of dry grasslands and the genetic diversity of several dry grassland plant species, therefore, in the context of habitat fragmentation and habitat conditions. Our study revealed a lack of correlation between species and genetic diversity. We demonstrated previously that SD mainly depends on habitat conditions (vegetation height and cover of litter), whereas GD is significantly affected by habitat fragmentation (distance to the nearest dry grassland in 1830 and connectivity in 2013). This seems to be the main reason why SD and GD are not congruent in fragmented grasslands. Our results support, hence, the observation that positive SGDCs can mainly be found in natural, island‐like study systems in equilibrium and at similar levels of heterogeneity. In fragmented dry grassland ecosystems, which differ in heterogeneity, this state of equilibrium may not have been reached mitigating the positive relationship between SD and GD. From our study, it can be concluded that in fragmented dry grasslands, the protection of SD does not necessarily ensure the conservation of GD.

## 1 INTRODUCTION

The relationship between species diversity (SD) and genetic diversity (GD) is one of the most fascinating topics in the field of ecological genetics, and it has already been postulated more than 40 years ago, that “the forces maintaining species diversity and genetic diversity are similar” (Antonovics, 1976). In the last decade, this “tenet” of ecological genetics (Antonovics, 2003) has been theoretically refined in several reviews (Vellend & Geber, 2005; Vellend et al., 2014) and practically analyzed in many studies using modeling (Vellend, 2005, 2006) or combining field and molecular approaches (Frey et al., 2015; He, Lamont, Krauss, Enright, & Miller, 2008; Pusças, Taberlet, & Choler, 2008; Taberlet et al., 2012).

Meanwhile the species‐genetic diversity correlation (SGDC) is generally considered to be positive (Vellend, 2005; Vellend et al., 2014), which is in line with the principles of the equilibrium theory of island biogeography (MacArthur & Wilson, 1967) and the island model of population genetics (Wright, 1940). It is assumed that both SD and GD respond similarly to the same local processes or that one level of biodiversity directly affects the other (Vellend, 2005). Local features of the habitat, such as area, isolation, and spatial or temporal heterogeneity affect migration, drift, and selection which have a comparable impact on species and alleles (Vellend & Geber, 2005).

Consequently, many studies dealing with the covariation of SD and GD reported positive SGDCs (Frey et al., 2015; He et al., 2008; Odat, Hellwig, Jetschke, & Fischer, 2009; Papadopoulou et al., 2011; Struebig et al., 2011; Wei & Jiang, 2012). Empirical data remain, however, contradictory since other studies exist revealing no positive relationship between SD and GD (Avolino & Smith, 2013; Odat, Jetschke, & Hellwig, 2004; Pusças et al., 2008; Silvertown, Biss, & Freeland, 2009; Taberlet et al., 2012).

Such a lack of correlation may result from a different response of SD and GD to environmental conditions. A decoupling of the two levels of biodiversity may for example result from a different response of SD and GD to historical range shifts and the spatial dynamics of suitable habitats (Pusças et al., 2008) or when the effects of habitat area and isolation on SD and GD are postponed temporarily. In systems which have not yet reached an equilibrium (Lamy et al., 2013), the covariation between SD and GD may therefore be nonsignificant (Taberlet et al., 2012).

Furthermore, environmental heterogeneity may have an impact on the positive relationship between SD and GD. Depending on a species’ traits, heterogeneity can decrease or increase population size (Evanno, Castella, Antoine, Paillat, & Goudet, 2009; Kahilainen, Puurtinen, & Kotiaho, 2014). If the amount of a specific habitat grows due to increasing habitat heterogeneity, the population sizes of species needing this kind of habitat also increase (Vellend, 2005). Consequently, the average amount of suitable area available for other species decreases which results in smaller population sizes of these species and reduces GD. Increased habitat heterogeneity may therefore lead to increasing SD while GD decreases simultaneously (Kahilainen et al., 2014). This means in other words, that the local abundance of a species can be related positively to its GD, but negatively with the abundance of other species (Lamy et al., 2013). A positive SGDC may in this way be mitigated by different levels of environmental heterogeneity.

Meanwhile, SGDCs have been investigated in many empirical studies (Avolino & Smith, 2013; Frey et al., 2015; He et al., 2008; Odat et al., 2009, 2004; Papadopoulou et al., 2011; Pusças et al., 2008; Silvertown et al., 2009; Struebig et al., 2011; Taberlet et al., 2012; Wehenkel, Bergmann, & Gregorius, 2006; Wei & Jiang, 2012). Most of these surveys are, however, based on genetic data of one species, only few SGDC studies included the genetic diversity of several species or larger species assemblages (Múrria et al., 2017; Múrria, Rugenski, Whiles, & Vogler, 2015; Papadopoulou et al., 2011). Since GD is also affected by species‐specific biological traits, multispecies approaches may reduce this bias and allow a more comprehensive genetic characterization (Múrria et al., 2017). Hence, there is a pronounced need for studies including the GD of several species and an ecological characterization of the analyzed habitat patches (Lamy et al., 2013). In the study presented here we chose, consequently, a multispecies approach to test whether species and genetic diversity are congruent in the nutrient‐poor and highly fragmented dry grasslands of southeastern Germany.

## 2 METHODS

**Figure 1:**
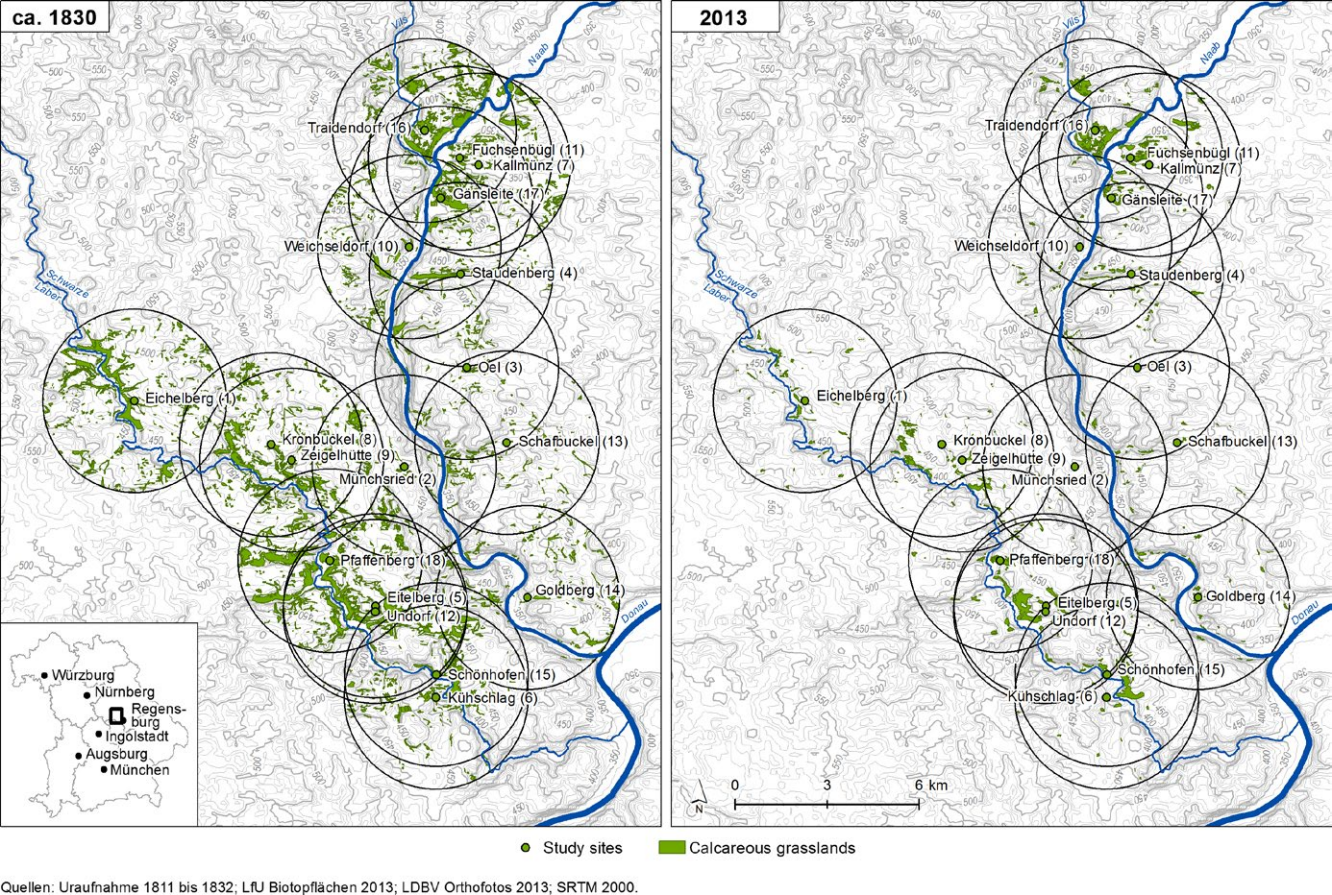
Geographic location of the 18 study sites in the valleys of the rivers Naab and Laber on the Franconian Alb in southeastern Germany near Regensburg (from Reisch et al., 2017)

Our study took place on 18 remnant dry grasslands in the valleys of the rivers Naab and Laber on the Franconian Alb in southeastern Germany near Regensburg (Figure 1). In this region, the dry grasslands date back at least to the Roman Empire period (Poschlod & Baumann, 2010) and are affected by afforestation, intensification, and abandonment since the 19th century. They have been grazed frequently until the 1960s, as reported for many other grasslands in central Europe (Poschlod, 2015), but are abandoned or infrequently grazed today.

Habitat fragmentation as well as habitat conditions of the selected dry grasslands and their impact on species and genetic diversity have already been analyzed separately in two previous studies (Huber, Huber, Stahl, Schmid, & Reisch, 2017; Reisch et al., 2017). For that, the selected study sites and all surrounding grasslands within a radius of three kilometers have been vectorized with a Geographic Information System (Arc Info 10.0, Esri) using aerial photos from 2013 and historical cadastral maps from 1830 (Figure 1). Based upon these data, we determined habitat area, distance to the nearest dry grassland within the 3‐km radius as well as connectivity among grasslands in 2013 and 1830. Moreover, we calculated habitat loss within the 3‐km radius since 1830 (Table 1). Habitat conditions were studied in ten study plots per site with s size of 2 × 2 m. For each plot, we reported vegetation height, cover of grass, litter, and bare soil. We took five soil samples at each study site and determined the phosphorous and potassium content, as well as the carbon‐to‐nitrogen ratio (Table 2). Using Bayesian regressions, the previous studies revealed different factors driving species and genetic diversity: SD strongly depended on vegetation height and cover of litter, whereas GD depended on the distance to the nearest dry grassland in 1830 and the connectivity in 2013 (Huber et al., 2017; Reisch et al., 2017).

**Table 1:** Habitat fragmentation data. Area of the selected study sites in m^2^ in 1830 and 2013 (HA_1830_ and HA_2013_), the distance to the nearest calcareous grassland in meter (D_1830_ and D_2013_), the connectivity of the grasslands (CO_1839_ and CO_2013_) within a radius of 3 km in 1830 and 2013, and the loss of calcareous grasslands within this radius since 1830 in % (HL)

St.	Name	HA_2013_	HA_1830_	D_2013_	D_1830_	CO_1830_	CO_2013_	HL
01	Eichelberg	445	715,410	58	129	100.90	8.35	83.01
02	Münchsried	631	0	980	133	23.89	2.34	79.12
03	Oel	763	6,725	391	81	9.76	1.96	61.88
04	Staudenberg	1,020	0	97	70	37.16	11.65	67.04
05	Eitelberg	1,399	0	117	97	130.80	44.65	84.25
06	Kühschlag	1,440	3,308	340	98	62.28	26.52	82.43
07	Kallmünz	1,546	11,072	175	168	82.16	48.29	83.75
08	Kronbuckel	1,695	1,176	382	62	59.55	13.94	80.55
09	Ziegelhütte	2,495	0	41	24	70.72	15.55	78.33
10	Weichseldorf	5,659	37,519	290	273	43.21	9.24	71.19
11	Fuchsenbügl	6,211	13,243	60	15	101.79	66.19	83.21
12	Undorf	8,009	0	91	59	132.94	50.75	84.49
13	Schafbuckel	12,033	17,338	150	192	13.19	3.60	77.90
14	Goldberg	22,160	0	32	121	21.15	7.19	66.83
15	Schönhofen	21,894	58,015	211	251	78.99	32.96	81.99
16	Traidendorf	24,405	134,710	58	44	97.91	71.44	84.95
17	Gänsleite	64,984	440,768	222	63	97.09	48.36	78.88
18	Pfaffenberg	91,067	631,523	87	94	183.16	38.90	85.32
	Mean	14,881	115,045	210	110	74.81	27.90	78.62
	SE	±5,828	±53,938	±53	±17	±11.01	±5.38	±1.67

**Table 2:** Habitat condition data. Habitat conditions of the selected study sites, described by the height of the vegetation in meter (VH), the cover of litter in % (CL), the cover of grass in % (CG), the proportion of bare soil in % (BS) as well as the content of phosphorous in mg/kg soil (P), potassium in mg/kg soil (K), and the ratio of carbon and nitrogen (C/N)

St.	Name	VH	CG	CL	BS	P	K	C/N
01	Eichelberg	1.18	92.8	23.0	0.0	14.70	369.53	18.3
02	Münchsried	0.95	62.5	19.0	0.3	15.66	101.22	13.7
03	Oel	0.94	90.0	24.0	0.0	36.48	232.81	15.6
04	Staudenberg	1.54	67.0	29.0	0.0	53.76	272.77	16.0
05	Eitelberg	1.08	88.2	16.6	0.0	14.18	130.26	22.6
06	Kühschlag	0.91	87.0	30.5	0.3	26.63	192.97	42.0
07	Kallmünz	0.77	82.5	15.0	5.5	12.70	195.62	17.6
08	Kronbuckel	1.13	88.8	10.3	0.4	23.85	220.63	16.6
09	Ziegelhütte	0.93	84.5	17.5	0.8	37.63	169.02	21.8
10	Weichseldorf	0.51	63.0	14.5	0.8	16.25	135.42	20.1
11	Fuchsenbügl	1.15	74.5	19.0	0.1	31.92	249.64	19.0
12	Undorf	1.13	90.3	25.5	0.1	41.19	173.90	37.4
13	Schafbuckel	1.01	78.0	07.7	0.0	37.90	240.98	18.1
14	Goldberg	1.13	62.0	29.0	0.2	37.57	127.73	19.9
15	Schönhofen	0.43	73.0	20.5	0.2	37.63	247.30	17.6
16	Traidendorf	0.31	48.0	38.0	1.5	09.62	319.02	13.9
17	Gänsleite	0.98	66.0	17.0	1.8	20.67	294.17	10.9
18	Pfaffenberg	0.65	78.0	10.4	0.5	08.04	126.00	11.1
	Mean	0.93	76.5	20.4	0.7	26.47	211.06	19.6
	SE	±0.1	±3.0	±1.9	±0.3	±3.11	±17.52	±1.9

In this study, we focus, in contrast to the previous investigations, on the correlation of SD and GD in a comparative approach. For reasons of comparability, we chose Simpson's Diversity and Nei's Gene diversity to measure SD and GD (Table 3), which are considered as analogous (He et al., 2008; Wei & Jiang, 2012) since they both follow the formula 1−∑p_i_^2^, where p_i_ is the frequency of the species or AFLP fragments. Simpson's Diversity was calculated based upon the occurrence of all plant species, which were recorded in the ten 2 × 2 m study plots per site. Because herbs are most sensitive to vegetation changes in dry grasslands, we recorded all herb species occurring at the study sites and within the plots. Since dry grassland specialists and other ecologically more generalist species may respond in different ways on environmental changes, we separated dry grassland specialists from the other species and determined Simpson's Diversity separately for all species (SD_all_) and for dry grassland specialists (SD_spec_). Species were considered as grassland specialists, if they belong to the phytosociological class Festuco‐Brometea following the flora of Oberdorfer (2001).

**Table 3:** Species diversity of the selected study sites was measured as Simpsons Diversity (SD) based upon all occurring species (SD_all_) and the grassland specialists (SD_spec_). Genetic diversity was estimated for five typical dry grassland species (GD_Pv_ *: Primula veris*, GD_Dc_: *Dianthus carthusianorum*, GD_Mf_: *Medicago falcata*, GD_Pc_: *Polygala comosa*, GD_Sp_: *Salvia pratensis*) as Nei's Gene Diversity using AFLPs. Based upon the values for the single species, we also calculated the mean genetic diversity over all species (GD_m_)

St.	Name	Species diversity		Genetic diversity					
		SD_all_	SD_spec_	GD_Pv_	GD_Dc_	GD_Mf_	GD_Pc_	GD_Sp_	GD_m_
01	Eichelberg	0.83	0.72	0.23	0.26	0.39	0.34	0.35	0.31
02	Münchsried	0.79	0.72	0.30	0.35	0.38	0.33	0.36	0.34
03	Oel	0.87	0.75	0.20	0.26	0.38	—	0.35	0.30
04	Staudenberg	0.82	0.58	0.20	0.25	0.36	—	0.37	0.30
05	Eitelberg	0.88	0.76	0.25	0.32	0.36	0.27	0.34	0.31
06	Kühschlag	0.85	0.77	0.27	0.32	0.36	0.31	0.33	0.32
07	Kallmünz	0.89	0.83	0.30	0.28	0.35	0.29	0.33	0.31
08	Kronbuckel	0.86	0.74	0.29	0.30	0.37	—	0.37	0.33
09	Ziegelhütte	0.86	0.81	0.30	0.31	0.38	0.36	0.36	0.34
10	Weichseldorf	0.86	0.81	0.22	0.29	0.37	0.33	0.34	0.31
11	Fuchsenbügl	0.82	0.73	0.22	0.29	0.37	0.31	0.35	0.31
12	Undorf	0.74	0.49	0.22	0.32	0.37	0.28	0.33	0.30
13	Schafbuckel	0.86	0.71	0.30	0.34	0.38	0.31	0.35	0.34
14	Goldberg	0.75	0.57	0.23	0.35	0.39	0.35	0.36	0.34
15	Schönhofen	0.83	0.75	0.26	0.31	0.36	0.29	0.33	0.31
16	Traidendorf	0.68	0.61	0.24	0.31	0.36	0.31	0.32	0.31
17	Gänsleite	0.81	0.72	0.27	0.31	0.38	0.31	0.34	0.32
18	Pfaffenberg	0.78	0.67	0.32	0.29	0.37	0.37	0.36	0.34
	Mean	0.71	0.82	0.18	0.23	0.29	0.25	0.26	0.32
	SE	±0.02	±0.01	±0.01	±0.01	±0	±0.01	±0.00	±0.00

Nei's Gene Diversity was calculated for each of the five typical dry grassland species *Primula veris *L., *Dianthus carthusianorum *L., *Medicago falcata* (L.) Arc., *Polygala comosa* Schkuhr, and *Salvia pratensis *L. using Amplified Fragment Length Polymorphisms (AFLPs). For the molecular analyses with a few exceptions (at three sites *P. comosa* could not be sampled), leaf material of 15 individuals per population and species was collected at all selected sites. The analysis comprised therefore 1,286 individuals from 87 populations in total.

Amplified Fragment Length Polymorphisms (AFLPs) were performed following the protocol from Beckmann Coulter as reported before (Bylebyl, Poschlod, & Reisch, 2008; Reisch, 2008). After a primer screening including 30 combinations, three primer combinations per species were chosen for the study (Reisch et al., 2017). AFLP products were separated by capillary gel electrophoresis on an automated sequencer (GeXP, Beckmann Coulter).

Results were checked using the GeXP software (Beckman Coulter) and analyzed using the software Bionumerics 4.6 (Applied Maths, Kortrijk, Belgium). From the computed gels, only those fragments that showed intense and articulate bands were taken into account for further analyses. Nineteen individuals were excluded from the analysis due to the lack of a clear banding pattern. Reproducibility of molecular analyses was investigated with 10% of all analyzed samples by means of estimating the genotyping error rate (Bonin et al., 2004), which was 3.8%. From the AFLP bands, a binary (0/1) matrix was created for each species. Based upon this matrix, we calculated Nei's Gene Diversity for each population of each species using the program AFLP SURV (Vekemans, 2002). Additionally, the mean genetic diversity for each study site over all species (GD_m_) was calculated as average of the values for the single species. Finally, the correlation between SD and GD was computed as the Pearson correlation coefficient for each single species and over all species.

## 3 RESULTS AND DISCUSSION

**Figure 2:**
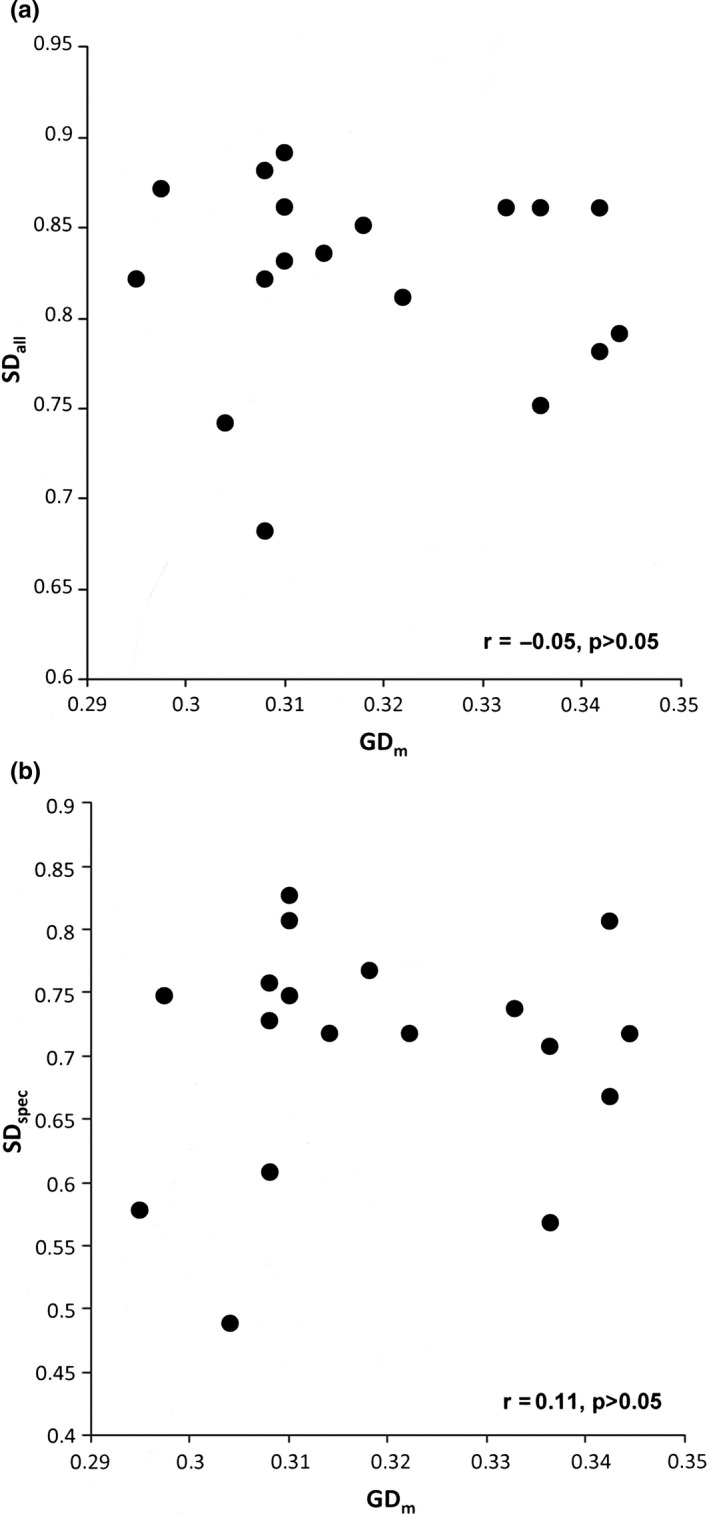
Relationship between species diversity (SD) and mean genetic diversity (GD_m_) for all species (a) and the grassland specialists (b). Correlations were not significant (*p* > 0.05)

Our study revealed no significant correlation between SD and GD, neither at the single species nor at the multispecies level (Table 4, Figure 2), although the correlation of species and genetic diversity (SGDC) is generally considered to be positive (Vellend, 2005; Vellend et al., 2014)—especially in isolated, patchy habitats (Whitlock, 2014). Under such conditions, both SD and GD depend on the area of the habitat fragment since increased drift in small fragments will cause the loss of rare species and alleles. In the same way, increased isolation of the habitat fragments will reduce SD and GD due to a lower level of immigration (Taberlet et al., 2012). SGDCs are therefore stronger when discrete sampling units such as habitat fragments are used, compared to nondiscrete sampling units such as equal area study plots (Vellend et al., 2014).

**Table 4:** Correlation of species diversity and genetic diversity using the Pearson correlation coefficient. Species diversity was measured as Simpson's diversity for all occurring species (SD_all_) and the grassland specialists (SD_spec_). Genetic diversity (GD) was estimated as Nei's Gene Diversity using AFLPs for five typical dry grassland species (GD_Pv_: *Primula veris*, GD_Dc_: *Dianthus carthusianorum*, GD_Mf_: *Medicago falcata*, GD_Pc_: *Polygala comosa*, GD_Sp_: *Salvia pratensis*). Based upon the values for the single species, we calculated the mean genetic diversity over all species (GD_m_). All correlations were not significant

	GD_Pv_	GD_Dc_	GD_Mf_	GD_Pc_	GD_Sp_	GD_m_
SD_all_	0.18	−0.30	−0.12	−0.16	0.18	−0.05
SD_spec_	0.39	−0.15	−0.15	0.20	−0.06	0.11

The nutrient‐poor dry grasslands, we analyzed in this study are highly fragmented (Huber et al., 2017; Reisch et al., 2017) and represent an island‐like study system. A strong and positive correlation of SD and GD could therefore be expected. However, in contrast to many previous studies (Frey et al., 2015; He et al., 2008; Odat et al., 2009; Papadopoulou et al., 2011; Struebig et al., 2011; Wei & Jiang, 2012), we observed that SD and GD are not congruent in the fragmented dry grasslands we analyzed here.

Such a lack of correlation may result from the different responses of SD and GD to the environmental conditions. Indeed, previous studies revealed different drivers for SD and GD. We already showed that SD strongly depends on vegetation height and cover of litter, which are directly related to the degree of grazing (Huber et al., 2017). Lack of grazing, due to abandonment, leads to the dominance of grasses and to the accumulation of biomass (Bobbink & Willems, 1987). This in turn causes ground shadowing and impedes germination (Jensen & Gutekunst, 2003; Piqueray et al., 2015). In consequence, especially the typical small dry grassland herbs disappear. In contrast, GD depends on the distance to the nearest dry grassland in 1830 and the connectivity in 2013 (Reisch et al., 2017). GD is therefore strongly affected by historical and present gene flow and drift via migration among the dry grassland fragments (Ouborg, Vergeer, & Mix, 2006). As a result genetic diversity is higher in populations from grasslands which were located close to other grasslands in 1830 and which exhibit higher levels of connectivity today. In the fragmented dry grasslands we analyzed here, SD is therefore mainly affected by the present land use, whereas GD is basically driven by historical and present landscape configuration.

A positive correlation of SD and GD would indicate that the mechanisms driving species and genetic diversity are effective simultaneously at both levels of biodiversity. The absence of such a correlation, as observed here, implies in contrast that different mechanisms are effective at the two organizational levels. It has already been demonstrated that a lack of correlation between SD and GD may especially occur when the effects derived from the equilibrium theory of island biogeography on SD and GD are postponed temporarily (Lamy et al., 2013). Delayed response of GD on fragmentation has been already reported for some species (Münzbergová et al., 2013; Vandepitte, Jacquemyn, Roldán‐Ruiz, & Honnay, 2007) and can most likely be connected to the persistence and the life span of the analyzed perennial plant species. Simulation experiments revealed that, under conditions of limited dispersal, historical landscape structure might be still detectable after more than 100 generations (Landguth et al., 2010). The observed lack of correlation between SD and GD may therefore indicate that the study system has not yet reached equilibrium (Lamy et al., 2013) and that a positive SGDC does not before the footprint of the historical landscape configuration is lost.

Moreover, the positive relationship between SD and GD may be concealed by different levels of habitat heterogeneity. Dry grasslands are semi‐natural ecosystems, which originated from grazing mainly by sheep (Poschlod & Wallis De Vries, 2002). It has already been shown that land use by grazing increases habitat heterogeneity (Marion, Bonis, & Bouzillé, 2010; Moinardeau, Mesléard, & Dutoit, 2016). In contrast, abandonment and the joint lack of grazing lead to a dominance of grasses and decrease habitat heterogeneity (Bobbink & Willems, 1987). Increasing habitat heterogeneity decreases the area available for the component species of a plant community and reduces, therefore, the size of the species’ populations. Since population size is positively correlated with GD (Leimu, Mutikainen, Koricheva, & Fischer, 2006), increased habitat heterogeneity may, therefore, lead to increasing SD while GD decreases simultaneously (Kahilainen et al., 2014). In a study on North American grasslands, it has recently been demonstrated that SD is positively linked to resource heterogeneity, whereas GD did not respond to this factor (Avolino & Smith, 2013). This supports our assumption that land use‐dependent levels of heterogeneity may contribute to the lack of correlation between SD and GD we observed here.

## 4 CONCLUSIONS

The conservation of biodiversity, as defined by the Convention on Biodiversity (CBD; www.cbd.int/convention/text/), requires the protection of variation at the level of ecosystems, species, and genes. Whereas ecosystems and species have been in the focus of conservation for a long time, much less attention has been paid to intraspecific genetic variation. From the mostly positive relationship between SD and GD, it has been derived that the protection of species may be attended by the conservation of genetic variation due to the parallel response of the two levels of biodiversity to environmental conditions (Kahilainen et al., 2014). It has even been suggested that genetic variation of common species predicts the occurrence of threatened species and that molecular analyses can be cost‐effective tools to identify areas with a large number of threatened species in conservation planning (Fuller et al., 2013). However, when SD and GD are not correlated, there can be a conflict between the conservation of species and conservation of their genetic variation (Kahilainen et al., 2014).

This is exactly true for the highly fragmented dry grasslands, we investigated in this study. They belong to the most species rich ecosystems in Europe and are, therefore, under a strong conservation focus (Poschlod & Wallis De Vries, 2002). Conservation authorities concentrated so far mainly on the protection of preferably large grasslands representing the typical short‐grass conditions to preserve as many species as possible. From our study, it can, however, be concluded that this approach does not automatically ensure also the preservation of genetic variation, since both levels of biodiversity are not correlated. In order to meet this objective, conservation strategies should also include dry grasslands, which have historically been located close to other dry grasslands and are currently well‐connected.

## CONFLICT OF INTEREST

The authors declare that they have no conflict of interest.

## AUTHOR CONTRIBUTION

CR and CS conducted the statistical analyses and wrote the manuscript.

## DATA ARCHIVING STATEMENT

Data are available from the Dryad Digital Repository: https://doi.org/10.5061/dryad.3127rs3.
